# Relationships between the *Osteocalcin* Gene Polymorphisms, Serum Osteocalcin Levels, and Hepatitis B Virus-Related Hepatocellular Carcinoma in a Chinese Population

**DOI:** 10.1371/journal.pone.0116479

**Published:** 2015-01-14

**Authors:** Yanqiong Liu, Liying Huang, Yu Lu, Xue-E Xi, Xiu-Li Huang, Qinghua Lu, Xiamei Huang, Shan Li, Xue Qin

**Affiliations:** 1 Department of Clinical Laboratory, First Affiliated Hospital of Guangxi Medical University, Nanning, Guangxi, China; 2 Department of Clinical Laboratory, Nanning Social Welfare Hospital, Nanning, Guangxi, China; University of North Carolina School of Medicine, UNITED STATES

## Abstract

**Background:**

Available evidence has demonstrated that osteocalcin may play a role in pathogenesis of cancer, and mutation of the *osteocalcin* gene may be involved in the cancer development. The aim of this study is to determine whether *osteocalcin* gene polymorphisms are associated with hepatitis B virus (HBV) related hepatocellular carcinoma (HCC) among Chinese population.

**Methods:**

A total of 515 subjects were divided into four groups: 129 patients with chronic hepatitis B (CHB), 62 patients with HBV-related liver cirrhosis (LC), 154 patients with HBV-related HCC, and 170 healthy controls. The polymerase chain reaction-restriction fragment length polymorphism strategy was used to detect *osteocalcin* gene *rs1800247* and *rs1543297* polymorphisms.

**Results:**

Compared with healthy controls, the *rs1800247* HH and Hh genotypes were associated with a significantly increased susceptibility to HCC (HH versus hh: OR = 6.828, 95% CI 2.620–17.795, *P* <0.001; Hh versus hh: OR = 6.306, 95% CI 3.480–11.423, *P* <0.001, respectively). Similarly, the subjects bearing the H allele of *rs1800247* had more than a 2.4-fold increased risk for development of HCC (OR = 2.484, 95% CI 1.747–3.532, *P* <0.001) compared with those bearing the h allele. In addition, we found significant decreased serum osteocalcin levels in HBV-related HCC patients (11.73±8.18 ng/mL) compared with healthy controls (15.3±6.06 ng/mL). Furthermore, the serum osteocalcin levels were significantly lower in HCC patients than healthy controls among the individuals with heterozygous Hh genotype (*P* = 0.003) and CT genotype (*P* <0.001). In contrast, there were no significant differences in the genotype and allele of *rs1543297* polymorphisms between the groups of patients and healthy controls.

**Conclusions:**

These findings for the first time suggest that genetic variant in *osteocalcin* gene *rs1800247* polymorphisms may be a risk factor for HBV-related HCC. We also find an inverse association of serum osteocalcin levels with HCC.

## Introduction

Hepatocellular carcinoma (HCC) is a common malignant neoplasm, with estimated 782,000 new cancer cases occurred in 2012 worldwide (50% in China alone) [1]. It ranks as the fifth most common incident cancer in men and the ninth in women [1]. Owing to its poor prognosis, it is the second commonest cause of death from cancer worldwide [1]. All of these data highlight the importance of a better understanding of risk factors related to HCC development. However, HCC is a multifactorial disease involving a complex interplay between genetic and environmental factors [2, 3]. It has been well recognized tha hepatitis B virus (HBV) and the hepatitis C virus (HCV) infection, smoking, aflatoxin exposure, and excess intake of alcohol are the major etiological factors accounting for HCC [4–6]. However, the molecular and cellular mechanisms for HCC pathogenesis remain largely elusive and need to be further elucidated. Association studies comparing genetic maker frequencies in patients and control groups without related diseases often implicate implicat a candidate gene in the etiology of a complex disease [7–9].

Osteocalcin, also called bone gamma-carboxyglutamate (gla) protein (BGLAP), is the most abundant noncollagenous protein component of bone. It involved in bone calcification, resorption, and remodeling [10, 11]. *Osteocalcin* gene is located at chromosome 1q25-q31. Recently, a polymorphism at nucleotide 298 in the promoter region of the *osteocalcin* gene at the restriction enzyme *Hind*III site was identified. The other one tagging single-nucleotide polymorphism (SNP) *rs1543294* (C/T) located 3’ of the *osteocalcin* gene and close to the polyamine-modulated factor 1 (PMF1) gene [12]. These two most commonly reported SNPs were expected to be important candidate sites of gene for bone mineral density and osteoporosis [12–17]. Our previous study had identified an inverse association of serum osteocalcin levels with metabolic syndrome in a Chinese population [18]. With respect to its relation to cancer, osteocalcin expression has been reported to associate with prostate cancer cell transformation [19]. Actually, the first report linking the osteocalcin and cancer was performed in 1988 by Francini et al. [20]. The authors reported that serum osteocalcin might be considered specific in the evaluation and monitoring of osteoblastic bone metastases in prostatic cancer [20]. Next to this, in 1989, Pietschmann et al. [21] reported that serum osteocalcin levels of patients with visceral metastases were significantly lower than in control subjects. After these, the positive roles of osteocalcin on breast and prostate cancers were further demonstrated [19, 22]. More recently, several studies demonstrated that lower serum osteocalcin levels were associated with the presence of non-alcoholic fatty liver disease, and also associated with serum transaminases and the extent of hepatocyte ballooning [23–25]. On the other hand, there was also study indicated that serum osteocalcin were significantly elevated in patients with HBV and HCV infections [26]. With regard to the genetic sutdies, Wu et al. [27] for the first time investigated the association between *osteocalcin* gene polymorphism and prostate cancer risk, and concluded that the *Hind*III polymorphism was a suitable genetic marker of prostate cancer.

However, to our knowledge, there has not been a study to examined the association between genetic polymorphisms in *osteocalcin* gene polymorphism and HBV-related HCC. The relationship between *osteocalcin* gene polymorphisms and the serum total osteocalcin levels remains unknown. The object of the present study is, for the first time, to determine whether *osteocalcin* gene *rs1800247* (*Hind*III) *and rs1543297* polymorphisms are associated with the susceptibility to chronic Hepatitis B (CHB), HBV-related liver cirrhosis (LC), and HBV-related HCC in a Chinese population.

## Subjects and Methods

### Study subjects

This was a hospital-based case-control study performed in a total of 515 unrelated subjects, including 129 patients with CHB (115 males, 14 females), 62 patients with HBV-induced LC (52 males, 10 females), 154 patients with HBV-related HCC (135 males, 19 females), and 170 healthy controls (147 males, 23 females) (Table 1). All the included patients with HBV-infected diseases were consecutively selected from the First Affiliated Hospital of Guangxi Medical University in Guangxi, China, between May and December 2013, which as described in detail previously [28–30]. All the included patients were confirmed to be positive for hepatitis B surface antigen (HBsAg) and hepatitis B virus core antibody (HbcAb) for at least six months.

**Table 1:** Demographic characteristics of the study population.

Variables	Controls (n = 170)	CHB (n = 129)	LC (n = 62)	HCC (n = 154)	P value
Age (years) mean ± SD	47.68±11.76	47.75±11.59	47.37±9.64	49.19±11.33	0.781
Sex *N* (%)					0.762
Male	147 (79.0)	115 (75.9)	52 (78.5)	135 (79.4)	
Female	23 (21.0)	14 (24.1)	10 (21.5)	19 (20.6)	
Smoking *N* (%)					0.319
Yes	127 (74.7)	85 (65.9)	42 (67.7)	113(73.4)	
No	43 (25.3)	44 (34.1)	20 (32.3)	41(26.6%)	
Drinking *N* (%)					0.194
Yes	131 (77.1)	92 (71.3)	51 (82.3)	108 (70.1)	
No	39 (22.9)	37 (28.7)	11 (17.7)	46 (29.9)	

*CHB* chronic hepatitis B, *HCC* hepatocellular carcinoma, *LC* liver cirrhosis, *SD* standard deviation, *IQR* interquartile range

As described in detail previously [9, 31], CHB was defined as positivity for HBsAg for a period of at least 6 months, serum HBV-DNA levers ≥1000 copies/mL, and elevated alanine aminotransferase (ALT) or aspartate aminotransferase (AST) (>40 IU/mL) at least once during the follow-up period. LC was diagnosed based on pathologic examination or typical morphologic findings from computed tomography (CT) or ultrasonography, and on the laboratory features. The diagnosis of HBV-related HCC was based on combination of clinical history, pathologic examination, imaging (CT, magnetic resonance imaging, or ultrasonography) and laboratory data, and/or histology. We only included the newly-diagnosed HCC patients; patients with other hepatitis virus infections, such as hepatitis C (HCV) or hepatitis E (HEV), or a medical history of HCC or other cancers were excluded. An alcohol drinker was defined as someone who consumed alcoholic beverages at least once per week for more than 6 months. Subjects were considered smokers if they smoked up to 1 year before the date of diagnosis for cases, or up to the date of interview for controls.

A total 170 controls without clinical evidence of hepatic disease or tumor were randomly selected from a pool of healthy volunteers who visited the general health check-up centers at the same hospitals during the same time period for routine scheduled physical exams. To control for the effects of potential confounders, controls were individually matched to cases based on sex, age (±5 years).

The study protocol was approved by the ethics committee of the First Affiliated Hospital of Guangxi Medical University. All of the involved patients and all healthy volunteers provided written informed consent.

### DNA extraction

Peripheral blood samples (2 mL) were collected from all of the subjects in ethylenediaminetetraacetic acid (EDTA)-coated vials and stored at −80°C until DNA extraction. Genomic DNA was extracted from white blood cell fractions using QIAamp DNA Blood Mini Kit (QIAGEN GmbH, Hilden, Germany), according to the manufacturer’s instructions. DNA concentration was determined spectrophotometrically.

### PCR amplification

The *rs1800247 and rs1543297* genotypes were performed using the polymerase chain reaction-restriction fragment length polymorphism (PCR-RFLP). The presence of the *rs1800247* polymorphisms was detected by amplifying genomic DNA with the following oligonucleotide primers: forward, 5’-CCGCAGCTCCCAACCACAATAAGCT-3’; and reverse, 5’-CAATAGGGCGAGGAGT-3’. For the *rs1543297*, the forward primer used was 5’-TGACCCCAAGAGGCTACAAG-3’; and forward primer used was 5’-CGGTAGCTGCCTAATCATGC-3’. The PCR reaction was performed in a total volume of 25 μl, consisting of 2 μl of genomic DNA, 1 μl of each primer, 12.5 μl of Green PCR Master Mix (Sangon Biotech, Shanghai, China), and 8.5 μl of nuclease-free double-distilled water.

For *rs1800247* genotype, the amplification protocol comprised initial denaturation at 95 C for 5 min; 30 cycles of denaturation at 94°C for 30 sec, annealing at 58°C for 30 sec, and extension at 72°C for 60 sec; and a final extension at 72° for 7 min. For *rs1543297* genotype, after an initial denaturation of 3 minutes’ duration at 95°, 30 cycles of denaturation (94°C, 45 sec), annealing (61°C, 30 sec), and elongation (72°C, 45 sec) were followed by a final elongation step at 72°C for 7 min.

### Polymorphism genotyping

**Figure 1:**
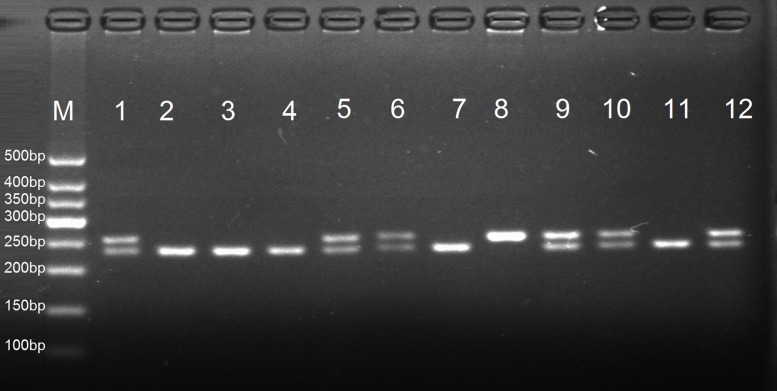
PCR-RFLP assay for analyzing the *rs1800247* polymorphisms of the *osteocalcin* gene. Lanes M: DNA Marker; Lanes 1, 5, 6, 9, and 10 show Hh genotype; Lanes 2, 3, 4, 7, and 11 show hh genotype; lane 8 shows HH genotype.

**Figure 2:**
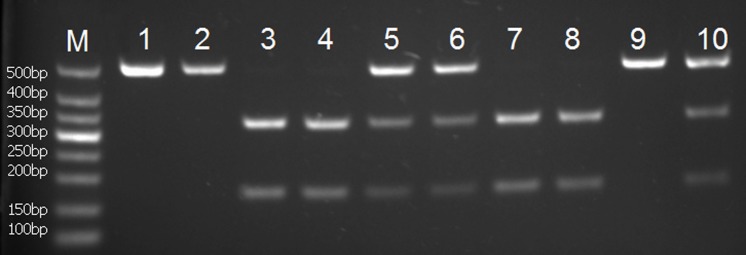
PCR-RFLP assay for analyzing the *rs1543297* polymorphisms of the *osteocalcin* gene. Lanes M: DNA Marker; Lanes 1, 2 and 9 show CC genotype; lane 5, 6, and 10 shows CT genotype; Lanes 3, 4, 7 and 8 show TT genotype.

For *rs1800247 and rs1543297*, 10 μl aliquots of the PCR products were digested at 37°C for 3 hours with 1 μl of *Hind*III or *Tai*I restriction enzymes, respectively. Digested fragments were separated by electrophoresis in 2% agarose gel containing ethidium bromide and the fragments, and visualized by the UV transilluminator. To control the quality of genotyping, negative control was performed in each genotyping assay. As a result, for *rs1800247* polymorphism, the homozygous HH genotype yielded 253bp products, heterozygous Hh genotype yielded 253bp and 232bp product, whereas homozygous hh allele yielded a 232bp product (Fig. 1). For *rs1543297* polymorphism, homozygous wild-type CC genotype yielded 502bp products, heterozygous CT genotype yielded 502bp, 330bp, and 172bp whereas homozygous mutant TT genotype yielded 330bp and 172bp products (Fig. 2).

### DNA sequencing

To confirm the accuracy of genotyping by PCR-RFLP, we selected 50 DNA samples (about 10%) at random and subjected them to direct DNA sequencing of PCR products with an ABI Prism 3100 (Applied Biosystems) in Shanghai Sangon Biological Engineering Technology & Services Co., Ltd., China. The results of DNA sequencing were 100% concordant (S1 Fig. and S2 Fig.).

### Serum osteocalcin determination

When blood samples were obtained, the serum was allowed to clot for 30 min at 4°C before centrifugation at 3000 rpm for 10 min at 4°C. Total serum was isolated and stored at −20°C until use. Total serum osteocalcin levels were with electrochemiluminescence immunoassay on COBAS 6000 system E601 (Elecsys module) immunoassay analyzer (Roche Diagnostics, GmbH, Mannheim, Germany) with the same batch of reagents. The intra- and interassay coefficients of variation were 3.8% and 4.5%, respectively.

### Statistical analysis

Normally distributed variables were presented as means and standard deviations (SD), whereas skewed variables were presented as median and interquartile range (IQR). One-way ANOVA was used to assess between-group variance of normally distributed variables. The Kruskal-Wallis test was used to compare skewed variables among the groups, and if significant, followed by Student-Newman-Keuls two-paired analyses. Frequency among the groups was compared using the χ^2^ test or Fisher’s exact test when appropriate. The genotype frequencies for the *osteocalcin* gene polymorphisms against Hardy-Weinberg ratios were assessed using the goodness of fit χ^2^-test. Odds ratios (ORs) and 95% confidence intervals (CIs) were calculated by binary logistic regression to assess the risk conferred by a particular allele and genotype by adjusted for gender, age, smoking, and drinking status. Haplotype analyses were performed using SHEsis software [32]. To elucidate the interaction of genotypes on osteocalcin serum levels, the data were analyzed by two-way analysis of variance for independent samples. All of the statistical analyses were performed in the Statistical Package for Social Sciences (SPSS, version 13.0). Statistical significance was assumed at two-sided values of *P* <0.05 level.

## Results

### Characteristics of the study population

The characteristics of the cases and the control subjects are shown in Table 1. The mean ages (SD) of the control group, CHB, LC and HCC group were 47.68±11.76, 47.75±11.59, 47.37±9.64, and 49.19±11.33, respectively. There were no significant differences for mean age, sex, smoking status, and alcohol consumption status between groups, which suggested the cases’ data are comparable with the controls’ (all *P* >0.05). In addition, we also did not found any factors, such as the endocrine condition and serum calcium concentration that significant influence the assessment of serum osteocalcin concentration in our included subjects, Furthermore, a Hardy–Weinberg equilibrium (HWE) test was performed for both investigated SNPs. The distribution of the *rs1800247* and *rs1543297* SNPs among controls was consistent with the Hardy-Weinberg equilibrium (*P* = 0.370 and 0.573, respectively)

### CHB patients versus healthy controls

The genotype and allele distributions of the *rs1800247* and *rs1543297* sites in the *osteocalcin* gene among the CHB cases and control subjects are described in Table 2. The frequencies of the hh, Hh, and HH genotypes of *rs1800247* were 48.8%, 44.1%, and 7.1% in healthy controls, and were 46.5%, 45.0%, and 8.5% in CHB patients, respectively. The frequencies of the CC, CT and TT genotypes of *rs1543297* were 12.9%, 48.8%, and 38.3% in healthy controls, and were 8.5%, 48.8%, and 43.0% in CHB patients, respectively. Binary logistic regression analyses adjusted for age, sex, smoking status, and alcohol consumption showed no significant differences between the genotype and allele frequencies of *rs1800247* and *rs1543297* polymorphisms and CHB risk, even after stratification of the study groups by gender. Haplotype analyses were performed in CHB patients and healthy controls using the SHEsis software and the possible two haplotype frequencies were observed. There were also no significant differences in the haplotype frequencies of the *osteocalcin* gene between this two study groups.

**Table 2:** Genotype and allele frequencies of two SNPs in the *osteocalcin* gene between CHB patients and healthy controls.

	**Overall**				**Females**				**Males**			
**Genotypes**	**Controls (n = 170)**	**CHB (n = 129)**	OR (95% CI)*	***p***	**Controls (n = 23)**	**CHB (n = 14)**	OR (95% CI)*	***p***	**Controls (n = 147)**	**CHB (n = 115)**	OR (95% CI)*	***p***
***rs1800247***												
hh	83(48.8)	60(46.5)	1.00^ref^		14(60.9)	6(42.9)	1.00^ref^		69(46.9)	54(47.0)	1.00^ref^	
Hh	75(44.1)	58(45.0)	1.068(0.662–1.724)	0.782	7(30.4)	7(50.0)	2.295(0.552–9.544)	0.238	68(46.3)	51(44.3)	0.959(0.496–3.320)	0.870
HH	12(7.1)	11(8.5)	1.257(0.518–3.050)	0.598	2(8.7)	1(7.1)	1.183(0.089–15.736)	0.907	10(6.8)	10(8.7)	1.284(0.496–3.320)	0.611
Dominant model^a^	87(51.2)	69(53.5)	1.094(0.691–1.733)	0.692	9(39.1)	8(57.1)	2.043(0.527–7.915)	0.286	78(53.1)	61(53.0)	1.000(0.613–1.633)	0.998
Recessive model^b^	158(92.9)	118(91.5)	1.227 (0.523–2.878)	0.637	21(91.3)	13(92.9)	0.808(0.066–9.821)	0.867	137(93.2)	105(91.3)	1.305(0.524–3.250)	0.567
h allele	241(70.9)	178(69.0)	1.00^ref^		35(76.1)	19(67.9)	1.00^ref^		206(70.1)	159(69.1)	1.00^ref^	
H allele	99(29.1)	80(31.0)	1.091(0.766–1.553)	0.617	11(23.9)	9(32.1)	1.502(0.529–4.267)	0.439	88(29.9)	71(30.9)	1.047(0.779–1.524)	0.817
***rs1543297***												
CC	22(12.9)	11(8.5)	1.00^ref^		1(4.3)	2(14.2)	1.00^ref^		21(14.3)	8(7.0)	1.00^ref^	
CT	83(48.8)	63(48.8)	1.677(0.740–3.799)	0.215	16(69.6)	6(42.9)	0.165(0.010–2.327)	0.170	67(45.6)	58(50.4)	2.233(0.919–5.428)	0.065
TT	65(38.3)	55(42.6)	1.868(0.815–4.283)	0.138	6(26.1)	6(42.9)	0.417(0.026–6.716)	0.605	59(40.1)	49(42.6)	2.180(0.887–5.355)	0.085
Dominant model^c^	148(87.1)	118(91.5)	1.762(0.803–3.869)	0.228	22(95.7)	12(85.8)	0.221(0.016–2.961)	0.283	126(85.7)	107(93.0)	2.208(0.940–5.189)	0.061
Recessive model^d^	105(61.8)	74(57.4)	1.201 (0.753–1.914)	0.442	17(73.9)	8(57.1)	2.125(0.519–8.699)	0.291	88(59.9)	64(56.6)	1.142(0.695–1.877)	0.601
C allele	127(37.3)	85(32.9)	1.00^ref^		18(39.1)	10(35.7)	1.00^ref^		109(37.1)	74(32.2)	1.00^ref^	
T allele	213(62.7)	173(67.1)	1.244(0.884–1.751)	0.264	28(60.9)	18(64.3)	1.108(0.411–2.987)	0.769	185(62.9)	156(67.8)	1.252(0.868–1.805)	0.243
Haplotype												
HC	127(37.4)	83(32.4)	0.805(0.572–1.132)	0.212								
HT	213(62.6)	173(67.6)	1.243(0.883–1.749)	0.212								

* Adjusted for sex, age, smoking and drinking by logistic regression model

^a^ Dominant model: HH+Hh versus hh;

^b^ Recessive model: HH versus hh+Hh;

^c^ Dominant model: TT+CT versus CC;

^d^ Recessive model: TT versus CC+CT.

### HBV-related LC patients versus healthy controls

The genotype and allele frequencies of the *rs1800247* and *rs1543297* sites in the *osteocalcin* gene among the HBV-related LC patients and healthy controls are shown in Table 3. Overall, we also did not found any significant differences of genotype and allele frequencies in *rs1800247* and *rs1543297* sites between HBV-related LC and control groups after adjusted for age, sex, smoking and drinking in binary logistic regression analyses. However, after stratification of the study groups by gender, we detected a statistically significant association of the *rs1800247* polymorphic Hh genotype and HBV-LC risk (OR = 9.611, 95% CI 1.236–74.756, *P* = 0.031) compared with the hh genotype in females. Under the dominant model, the combined genotype HH + Hh appeared to have higher susceptibility to HBV-LC in females (OR = 7.731, 95% CI 1.085–55.114, *p* = 0.041). The haplotypes were not associated with risk of HBV-LC.

**Table 3:** Genotype and allele frequencies of two SNPs in the *osteocalcin* gene between LC patients and healthy controls.

	**Overall**				**Females**				**Males**			
**Genotypes**	**Controls (n = 170))**	**LC (n = 62)**	OR (95% CI)*	***p***	**Controls (n = 23)**	**LC (n = 10)**	OR (95% CI)*	***p***	**Controls (n = 147)**	**LC (n = 52)**	OR (95% CI)*	***p***
***rs1800247***												
hh	83(48.8)	25(40.3)	1.00^ref^		14(60.9)	2(20.0)	1.00^ref^		69(46.9)	23(44.2)	1.00^ref^	
Hh	75(44.1)	30(48.4)	1.359(0.715–2.581)	0.366	7(30.4)	7(70.0)	9.611(1.236–74.756)	0.031	68(46.3)	23(44.2)	1.033(0.518–2.061)	0.966
HH	12(7.1)	7(11.3)	2.567(0.875–7.533)	0.205	2(8.7)	1(10.0)	2.829(0.121–66.012)	0.364	10(6.8)	6(11.5)	2.449(0.772–7.773)	0.298
Dominant model^a^	87(51.2)	37(59.7)	1.500(0.812–2.773)	0.251	9(39.1)	8(80.0)	7.731(1.085–55.114)	0.041	78(53.1)	29(55.8)	1.185(0.614–2.286)	0.736
Recessive model^b^	158(92.9)	55(88.7)	1.676 (0.628–4.471)	0.298	21(91.3)	9(90.0)	1.167(0.093–14.562)	0.905	137(93.2)	46(88.5)	1.787(0.616–5.188)	0.280
h allele	241(70.9)	80(64.5)	1.00^ref^		35(76.1)	11(55.0)	1.00^ref^		206(70.1)	69(66.3)	1.00^ref^	
H allele	99(29.1)	44(35.5)	1.461(0.929–2.294)	0.189	11(23.9)	9(45.0)	2.703(0.804–9.078)	0.087	88(29.9)	35(33.7)	1.303(0.798–2.128)	0.480
***rs1543297***												
CC	22(12.9)	9(14.5)	1.00^ref^		1(4.3)	1(10.0)	1.00^ref^		21(14.3)	8(15.4)	1.00^ref^	
CT	83(48.8)	32(51.6)	1.023(0.415–2.524)	0.894	16(69.6)	5(50.0)	0.470(0.023–9.762)	0.420	67(45.6)	27(51.9)	1.088(0.420–2.824)	0.906
TT	65(38.3)	21(33.9)	0.679(0.262–1.760)	0.614	6(26.1)	4(40.0)	1.165(0.048–28.093)	0.793	59(40.1)	17(32.7)	0.598(0.217–1.645)	0.575
Dominant model^c^	148(87.1)	53(85.5)	0.863(0.365–2.042)	0.755	22(95.7)	9(90.0)	0.629(0.033–12.065)	0.532	126(85.7)	44(84.6)	0.847(0.342–2.096)	0.847
Recessive model^d^	105(61.8)	41(66.1)	0.827(0.449–1.523)	0.542	17(73.9)	6(60.0)	1.889(0.393–9.085)	0.424	88(59.9)	35(67.3)	0.724(0.372–1.411)	0.342
C allele	127(37.3)	50(40.3)	1.00^ref^		18(39.1)	7(35.0)	1.00^ref^		109(37.1)	43(41.3)	1.00^ref^	
T allele	213(62.7)	74(59.7)	0.789(0.510–1.221)	0.550	28(60.9)	13(65.0)	1.445(0.442–4.730)	0.751	185(62.9)	61(58.7)	0.723(0.451–1.158)	0.441
Haplotype												
HC	127(37.4)	50 (40.3)	1.133(0.744–1.726)	0.560								
HT	213(62.6)	74 (62.6)	0.882(0.579–1.344)	0.560								

* Adjusted for sex, age, smoking and drinking by logistic regression model

^a^ Dominant model: HH+Hh versus hh;

^b^ Recessive model: HH versus hh+Hh;

^c^ Dominant model: TT+CT versus CC;

^d^ Recessive model: TT versus CC+CT.

### HBV-related HCC patients versus healthy controls

The genotype and allele frequencies of the *rs1800247* and *rs1543297* sites in the *osteocalcin* gene among the HBV-related HCC patients and healthy controls are shown in Table 4. In the binary logistic regression analyses, the *rs1800247* Hh and HH genotypes were associated with a significantly increased risk of HCC compared with the hh genotype (OR = 6.306, 95% CI 3.480–11.423, *P* < 0.001 and OR = 6.828, 95% CI 2.620–17.795, *P*< 0.001). The data also revealed that subjects with the H allele appeared to have more than a 2.4-fold increased risk for the development of HCC compared with those bearing the h allele (OR = 2.484, 95% CI 1.747–3.532, *P*< 0.001). Under the dominant model, the combined genotype HH + Hh appeared to have higher susceptibility to HCC (OR = 6.403, 95% CI 3.570–11.483, *P*< 0.001). The data also revealed that the *rs1543297* polymorphisms were not associated with HCC risk.

**Table 4:** Genotype and allele frequencies of two SNPs in the *osteocalcin* gene between HCC patients and healthy controls.

	**Overall**				**Females**				**Males**			
**Genotypes**	**Controls (n = 170)**	**HCC (n = 154)**	OR (95% CI)*	***p***	**Controls (n = 23)**	**HCC (n = 19)**	OR (95% CI)*	***p***	**Controls (n = 147)**	**HCC (n = 135)**	OR (95% CI)*	***p***
***rs1800247***												
hh	83(48.8)	21(13.6)	1.00^ref^		14(60.9)	3(15.8)	1.00^ref^		69(46.9)	18(13.3)	1.00^ref^	
Hh	75(44.1)	118(76.6)	6.306(3.480–11.423)	<0.001	7(30.4)	15(78.9)	13.718(2.248–83.719)	0.005	68(46.3)	103(76.3)	5.835(3.088–11.024)	<0.001
HH	12(7.1)	15(9.8)	6.828(2.620–17.795)	<0.001	2(8.7)	1(5.3)	1.887(0.090–39.610)	0.531	10(6.8)	14(10.4)	7.823(2.808–21.790)	<0.001
Dominant model^a^	87(51.2)	133(86.4)	6.403(3.570–11.483)	<0.001	9(39.1)	16(84.2)	10.295(1.842–57.545)	0.008	78(53.1)	117(86.7)	6.095(3.259–11.399)	<0.001
Recessive model^b^	158 (92.9)	139 (90.2)	1.421 (0.643–3.139)	0.383	21(91.3)	18(94.7)	1.167 (0.068–20.016)	0.915	137(93.2)	121(89.6)	1.585(0.679–3.700)	0.283
h allele	241(70.9)	160(51.9)	1.00^ref^		35(76.1)	21(55.3)	1.00^ref^		206(70.1)	139(51.5)	1.00^ref^	
H allele	99(29.1)	148(48.1)	2.484(1.747–3.532)	<0.001	11(23.9)	17(44.7)	2.673(0.940–7.603)	0.054	88(29.9)	131(48.5)	2.464(1.695–3.583)	<0.001
***rs1543297***												
CC	22(12.9)	23(14.9)	1.00^ref^		1(4.3)	5(26.3)	1.00^ref^		21(14.3)	18(13.3)	1.00^ref^	
CT	83(48.8)	71(46.1)	0.875(0.431–1.778)	0.554	16(69.6)	7(36.8)	0.132(0.012–1.492)	0.102	67(45.6)	64(47.4)	1.133(0.528–2.431)	0.767
TT	65(38.3)	60(39.0)	0.701(0.336–1.462)	0.720	6(26.1)	7(36.8)	0.408(0.031–5.362)	0.216	59(40.1)	53(39.3)	0.761(0.346–1.674)	0.900
Dominant model^c^	148(87.1)	131(85.1)	0.796(0.407–1.559)	0.604	22(95.7)	14(73.7)	0.196(0.019–2.041)	0.114	126(85.7)	117(86.7)	0.953(0.463–1.961)	0.817
Recessive model^d^	105 (61.2)	94 (61.0)	1.031 (0.659–1.614)	0.893	17(73.9)	12(63.2)	1.653(0.443–6.170)	0.453	88(59.9)	82(60.7)	0.964 (0.598–1.554)	0.881
C allele	127(37.3)	117(38.0)	1.00^ref^		18(39.1)	17(44.7)	1.00^ref^		109(37.1)	100(37.0)	1.00^ref^	
T allele	213(62.7)	191(62.0)	0.830(0.588–1.170)	0.846	28(60.9)	21(55.3)	0.962(0.360–2.574)	0.604	185(62.9)	170(63.0)	0.821(0.568–1.187)	0.993
Haplotype												
HC	127(37.4)	117(38.0)	1.027(0.747–1.424)	0.868								
HT	213(62.6)	191(62.0)	0.973(0.708–1.338)	0.868								

* Adjusted for sex, age, smoking and drinking by logistic regression model

^a^ Dominant model: HH+Hh versus hh;

^b^ Recessive model: HH versus hh+Hh

^c^ Dominant model: TT+CT versus CC;

^d^ Recessive model: TT versus CC+CT

After stratification of the study groups by gender, the date showed that the *rs1800247* Hh, HH genotypes and H allele were all associated with a significantly increased risk of HCC compared with the hh genotype in males. However, in female subjects, comparing with the hh genotype, the Hh genotype was significantly related to an increased risk of HCC after adjusted by age, sex, smoking and drinking status using binary logistic regression analyses (OR = 13.718, 95% CI 2.248–83.719, *P* = 0.005), but no significant differences were found in the HH genotype and H allele. Regarding the *rs1543297* SNP, we also found no significant differences of the genotype and allele frequencies between HCC patients and controls in both female and male subjects.

The genotypes and alleles frequencies of the SNPs in our control group were compared with those from different races the Haplotype Map (HapMap) Project (http://www.ncbi.nlm.nih.gov/snp/). There was lack of Hapmap data of the SNP *rs1800247*. Thus, we only compared gene frequencies of controls for *rs1543297* with those from the *Hapmap* database (S1 Table). The data suggests that the distribution of the SNP *rs11549465* among the healthy controls in the present study was similar to that in HCB (Han Chinese in Beijing), JPT (Japanese in Tokyo), and YRI (Yoruba in Ibadan) populations, but was significantly different from that in CEU (Utah residents with northern and western European ancestry) population. For the *rs1543297* polymorphism, the frequencies of genotype CC (63.7%) and allele C (80.5%) in CEU population are significantly higher than those (genotype CC: 12.9%, allele C 37.3%) in our present study.

### Serum osteocalcin levels

Serum samples were available for 57controls, 83 CHB cases, 49 LC cases, and 125 cases. Osteocalcin values showed a markedly skewed distribution, thus the mean osteocalcin values were expressed as the median ± inter-quartile range (Table 5). The mean serum osteocalcin concentration in healthy controls, CHB patients, LC patients, and HCC patients was 15.3±6.06 ng/mL, 13.3±8.38 ng/mL, 14.57±9.53 ng/mL, and 11.73±8.180 ng/mL, respectively. The normal reference interval of osteocalcin levels in healthy Chinese are 12.49–43.94 ng/mL [33]. The serum osteocalcin levels of the controls in our study were within the normal reference range of osteocalcin levels in healthy Chinese. There were significant differences in serum osteocalcin levels among groups using the Kruskal-Wallis test (p < 0.001). And then, two paired analyses were performed by Student-Newman-Keuls test. The data revealed that serum osteocalcin concentration was significant decreased in HBV-related HCC patients when compared with the healthy controls, CHB patients, and LC patients. But there was no significant difference of serum osteocalcin concentration between LC patients and healthy controls.

**Table 5:** Association of *osteocalcin* polymorphisms with serum osteocalcin levels (median ± IQR, ng/mL) in cases and healthy controls.

	**Overall**		***rs1800247* genotype**							***rs1543297* genotype**						
**Groups**			hh		Hh		HH			CC		CT		TT		
	**N**	**OC levels**	**N**	**OC levels**	**N**	**OC levels**	**N**	**OC levels**	p value*	**N**	**OC levels**	**N**	**OC levels**	**N**	**OC levels**	p value*
Controls	57	15.3±6.06	28	14.92±5.34	27	15.41±7.79	2	16.75±8.82	0.169	5	15.1±6.13	33	15.48±6.57	19	14.58±7.89	0.098
CHB cases	83	13.3±8.38	38	14.49±7.31	37	13.25±7.81	8	14.86±5.02	0.618	10	14.86±5.86	38	13.62±8.09	35	13.25±8.74	0.987
LC cases	49	14.57±9.53	22	15.48±9.43	22	14.76±10.13	5	13.25±6.39	0.858	8	17.29±17.51	23	17.35±8.9	18	14.35±8.07	0.689
HCC cases	125	11.73±8.18	18	12.66±8.04	94	11.59±7.62	13	10.11±9.11	0.822	17	10.61±7.41	60	11.56±8.73	48	13.07±7.72	0.831
p value**	<0.001		0.567		0.003		0.162		-	0.291		<0.001		0.416		-

*CHB* chronic hepatitis B, *HCC* hepatocellular carcinoma,*LC* liver cirrhosis,*IQR* interquartile range, OC, osteocalcin, *N* group number

* Kruskal-Wallis test: comparing the difference of serum osteocalcin levels in the three genotypes among the same group subjects.

** Kruskal-Wallis test: comparing the difference of serum osteocalcin levels in the four group subjects among the individuals with the same genotype.

### Association of osteocalcin gene polymorphisms and osteocalcin levels

As shown in Table 5, we found no significant association between the *osteocalcin* gene *rs1800247* and *rs1543297* polymorphisms and serum osteocalcin levels in healthy controls, CHB patients, HBV-related LC patients, as well as HCC patients group (*P*> 0.05). However, when compared difference of serum osteocalcin levels in these four groups subjects among the individuals with same genotype, it demonstrated that the serum osteocalcin levels were significantly lower in HCC patients than healthy controls among the individuals with heterozygous Hh genotype (*P* = 0.003) and CT genotype (*P*<0.001 ).

## Discussion

In the present study, we perform a large case-control study in a Chinese population to investigate whether *osteocalcin* gene polymorphisms are associated with the occurrence of CHB, HBV-related LC, and HBV-related HCC. In addition, we also examine whether the *osteocalcin* gene polymorphisms correlate with serum total osteocalcin levels. To the best of our knowledge, this is the first study conducted on the association between the *osteocalcin* gene polymorphisms, serum osteocalcin levels and the susceptibility to HBV-related disease patients. The present results revealed that the HH and Hh genotypes of the *rs1800247* (*Hind*III) site in the *osteocalcin* gene were associated with a significantly increased susceptibility to HBV-related HCC compared with the hh genotype after adjustment for age, sex, smoking status, and alcohol consumption (OR = 6.828, 95% CI 2.620–17.795, *P*< 0.001; OR = 6.306, 95% CI 3.480–11.423, *P*< 0.001, respectively). Similarly, the subjects bearing the H allele of *rs1800247* polymorphism also had more than 2.4-fold (OR = 2.484, 95% CI 1.747–3.532) increased risk for the development of HCC compared with those bearing the h allele. Further classifying the subjects into subgroups based on gender, the direction and magnitude of risk did not change. However, in this study, genotype and allele frequencies of *rs1543297* polymorphism and haplotypes of the *osteocalcin* gene in HBV-related patients were not significantly different from those in healthy controls. Furthermore, we found that the serum osteocalcin levels of patients with HBV-related HCC were significantly lower than in the overall healthy control subjects, and in the individuals with heterozygous Hh genotype (*P* = 0.003) and CT genotype (*P*<0.001). These results indicated that the *osteocalcin* gene *rs1800247* polymorphism might serve as a candidate genetic marker for screening for HBV-related HCC.

Osteocalcin is synthesized by mature osteoblasts and regulated by vitamin D and parathyroid hormone. It is a biomarker for bone-formation activity [34], and serum osteocalcin also can be considered a marker of bone turnover [35]. Osteocalcin is also related to bone resorption and may change blood levels of calcium ions [36]. Therefore, it seems reasonable to hypothesis that osteocalcin might correlate with cancer progression. Osteocalcin gene is located at chromosome 1q25-q31. Genetic polymorphisms in the *osteocalcin* gene may affect osteocalcin production or protein expression, thus modulate cancer risk. The *Hind*III marker represents a C→T transition in the promotor region of the *osteocalcin* gene and is of potential functional importance in the regulation of the *osteocalcin* gene expression [15]. Recently, the studies related the positive roles of osteocalcin on cancer mainly focus on prostate and breast cancer [20–22, 27, 37, 38]. In this study, we tested the association between the *osteocalcin* gene polymorphism and HBV-related liver diseases in 512 Chinese subjects. Our results may provide support for the importance of osteocalcin in the pathogenesis of HBV-related HCC. However, the exact mechanism as to how *osteocalcin* polymorphisms affect HCC risk is still unknown, thus our findings need to be confirmed by further larger studies, in which the mechanisms underlying this association also should be directed.

In the present study, we observed that the *rs1800247* HH genotype and H allele in *osteocalcin* gene were associated with a significantly increased risk of HCC compared with the hh genotype in male subjects but not in female subjects. The difference between male and female in our analyses was unexpected and difficult to explain. One of the potential explanations may be the result of the larger total number of subjects in the male HCC group (n = 135) than in the female HCC group (n = 19). With a larger sample size, increased statistical power could be obtained. Therefore, a great confusion has arisen regarding the sex difference in HCC response to *rs1800247* polymorphism, and these findings need to be confirmed by further larger studies.

In this study, we found that the serum osteocalcin levels were significantly lower in HBV-related HCC patients compared with the healthy controls, CHB patients, and LC patients. The results of our study suggest that a depressed osteocalcin response might play a role in HBV-related HCC etiology. In addition, we demonstrated that the serum osteocalcin levels were significantly reduced in HCC patients than healthy controls among individuals with *rs1800247* heterozygous Hh genotypes. Our results suggested that the *osteocalcin* gene polymorphism may contribute to an increased HBV-related HCC risk through regulate the expression of serum osteocalcin levels. But the mechanisms underlying the antitumor activity of osteocalcin are not clearly understood. Further studies are needed to direct the molecular mechanisms by which osteocalcin is involved in susceptibility to HBV-related HCC.

However, several potential limitations of this study must be acknowledged. First, our patient sample size was not large enough. Our data may be of limited value and therefore, an additional study with more subjects is expected. Second, our included subjects were recruited from only one hospital in Guangxi and were limited to only one ethnic population. Our results may not be generalized to other populations and not be well representative of the entire target population. However, we have tried our best to reduce the selection bias to the lowest possible level by matching the age, gender, smoking and drinking status between the cases and controls. Third, our research investigated only two SNPs polymorphisms in the *osteocalcin* gene. It would be interesting to identify more SNPs polymorphisms in the *osteocalcin* gene and study their correlations with HBV-related HCC. Thus, the results of this research must be interpreted cautiously in light of the limitations.

In conclusion, our results identify that the *Hind*III (*rs1800247*) SNP variant in *osteocalcin* gene is associated with significantly increased susceptibility to HBV-related HCC in Guangxi Chinese populations. In addition, we found an inverse association between osteocalcin levels and HBV-related HCC. These results may provide support for the importance of *osteocalcin* gene in the pathogenesis of HBV-related HCC.

## Supporting Information

S1 Fig Sequencing map of the genotypes for the rs1800247 polymorphisms of the *osteocalcin* gene. Arrow in parts a–c indicates (hh) CC, (Hh) CT and (HH) TT genotypes, respectively.
(TIF)

S2 Fig Sequencing map of the genotypes for the rs1543297 polymorphisms of the *osteocalcin* gene. Arrow in parts a–c indicates CC, CT and TT genotypes, respectively.
(TIF)

S1 Table Comparison of genotype and allele frequencies in the healthy control subjects of our study and that from the HapMap project. (DOCX)

## References

[1] Ferlay J, Soerjomataram I, Ervik M, et al (2013) GLOBOCAN 2012 v1.0, Cancer Incidence and Mortality Worldwide: IARC CancerBase No. 11 [Internet]. Lyon, France: International Agency for Research on Cancer. Available: http://globocan.iarc.fr. Accessed 2014 May 15.

[2] Aravalli RN, Cressman EN, Steer CJ (2013) Cellular and molecular mechanisms of hepatocellular carcinoma: an update. Arch Toxicol 87: 227–247. 10.1007/s00204-012-0931-2 23007558

[3] Mathew S, Ali A, Abdel-Hafiz H, Fatima K, Suhail M, et al (2014) Biomarkers for virus-induced hepatocellular carcinoma (HCC). Infect Genet Evol 26C: 327–339. 10.1016/j.meegid.2014.06.014 24956436

[4] Farazi PA, DePinho RA (2006) Hepatocellular carcinoma pathogenesis: from genes to environment. Nat Rev Cancer 6: 674–687. 10.1038/nrc1934 16929323

[5] Chuang SC, La Vecchia C, Boffetta P (2009) Liver cancer: Descriptive epidemiology and risk factors other than HBV and HCV infection. Cancer Letters 286: 9–14. 10.1016/j.canlet.2008.10.040 19091458

[6] Chen CJ, Yu MW, Liaw YF (1997) Epidemiological characteristics and risk factors of hepatocellular carcinoma. J Gastroenterol Hepatol 12: S294–308. 10.1111/j.1440-1746.1997.tb00513.x 9407350

[7] Elston RC (1998) Linkage and association. Genet Epidemiol 15: 565–576. 10.1002/(SICI)1098-2272(1998)15:6<565::AID-GEPI2>3.0.CO;2-J 9811419

[8] Li S, Deng Y, Chen ZP, Huang S, Liao XC, et al (2011) Genetic polymorphism of interleukin-16 influences susceptibility to HBV-related hepatocellular carcinoma in a Chinese population. Infect Genet Evol 11: 2083–2088. 10.1016/j.meegid.2011.09.025 22019522

[9] Liu Y, Liu Y, Huang X, Sui J, Mo C, et al (2014) Association of PvuII and XbaI polymorphisms in estrogen receptor alpha gene with the risk of hepatitis B virus infection in the Guangxi Zhuang population. Infect Genet Evol 27C: 69–76. 10.1016/j.meegid.2014.07.002 25014269

[10] Sugiyama T, Kawai S (2001) Carboxylation of osteocalcin may be related to bone quality: a possible mechanism of bone fracture prevention by vitamin K. J Bone Miner Metab 19: 146–149. 10.1007/s007740170034 11368299

[11] Lian JB, Gundberg CM (1988) Osteocalcin. Biochemical considerations and clinical applications. Clin Orthop Relat Res: 267–291. 3275514

[12] McGuigan F, Kumar J, Ivaska KK, Obrant KJ, Gerdhem P, et al (2010) Osteocalcin Gene Polymorphisms Influence Concentration of Serum Osteocalcin and Enhance Fracture Identification. Journal of Bone and Mineral Research 25: 1392–1399. 10.1002/jbmr.32 20200947

[13] Chen HY, Tsai HD, Chen WC, Wu JY, Tsai FJ, et al (2001) Relation of polymorphism in the promotor region for the human osteocalcin gene to bone mineral density and occurrence of osteoporosis in postmenopausal Chinese women in Taiwan. J Clin Lab Anal 15: 251–255. 10.1002/jcla.1036 11574953 PMC6808128

[14] Raymond MH, Schutte BC, Torner JC, Burns TL, Willing MC (1999) Osteocalcin: genetic and physical mapping of the human gene BGLAP and its potential role in postmenopausal osteoporosis. Genomics 60: 210–217. 10.1006/geno.1999.5893 10486212

[15] Dohi Y, Iki M, Ohgushi H, Gojo S, Tabata S, et al (1998) A novel polymorphism in the promoter region for the human osteocalcin gene: the possibility of a correlation with bone mineral density in postmenopausal Japanese women. J Bone Miner Res 13: 1633–1639. 10.1359/jbmr.1998.13.10.1633 9783552

[16] Yamada Y, Ando F, Niino N, Shimokata H (2003) Association of polymorphisms of interleukin-6, osteocalcin, and vitamin D receptor genes, alone or in combination, with bone mineral density in community-dwelling Japanese women and men. J Clin Endocrinol Metab 88: 3372–3378. 10.1210/jc.2002-021449 12843190

[17] Kim JG, Ku SY, Lee DO, Jee BC, Suh CS, et al (2006) Relationship of osteocalcin and matrix Gla protein gene polymorphisms to serum osteocalcin levels and bone mineral density in postmenopausal Korean women. Menopause 13: 467–473. 16735944 10.1097/01.gme.0000182803.06762.fb

[18] Tan A, Gao Y, Yang X, Zhang H, Qin X, et al (2011) Low serum osteocalcin level is a potential marker for metabolic syndrome: results from a Chinese male population survey. Metabolism 60: 1186–1192. 10.1016/j.metabol.2011.01.002 21353261

[19] Koeneman KS, Kao C, Ko SC, Yang L, Wada Y, et al (2000) Osteocalcin-directed gene therapy for prostate-cancer bone metastasis. World J Urol 18: 102–110. 10.1007/s003450050181 10854144

[20] Francini G, Bigazzi S, Leone V, Gennari C (1988) Serum osteocalcin concentration in patients with prostatic cancer. Am J Clin Oncol 11 Suppl 2: S83–87. 10.1097/00000421-198801102-00021 3266542

[21] Pietschmann P, Zielinski C, Woloszczuk W (1989) Serum osteocalcin levels in breast cancer patients. J Cancer Res Clin Oncol 115: 456–458. 10.1007/BF00393337 2808485 PMC12211687

[22] Kamby C, Egsmose C, Soletormos G, Dombernowsky P (1993) The diagnostic and prognostic value of serum bone Gla protein (osteocalcin) in patients with recurrent breast cancer. Scand J Clin Lab Invest 53: 439–446. 10.3109/00365519309092538 8210965

[23] Liu JJ, Chen YY, Mo ZN, Tian GX, Tan AH, et al (2013) Relationship between Serum Osteocalcin Levels and Non-Alcoholic Fatty Liver Disease in Adult Males, South China. International Journal of Molecular Sciences 14: 19782–19791. 10.3390/ijms141019782 24084725 PMC3821586

[24] Yilmaz Y, Kurt R, Eren F, Imeryuz N (2011) Serum osteocalcin levels in patients with nonalcoholic fatty liver disease: Association with ballooning degeneration. Scandinavian Journal of Clinical & Laboratory Investigation 71: 631–636. 10.3109/00365513.2011.604427 21859358

[25] Aller R, Castrillon JLP, de Luis DA, Conde R, Izaola O, et al (2011) Relation of osteocalcin with insulin resistance and histopathological changes of non alcoholic fatty liver disease. Annals of Hepatology 10: 50–55. 21301010

[26] Mahdy KA, Ahmed HH, Mannaa F, Abdel-Shaheed A (2007) Clinical benefits of biochemical markers of bone turnover in Egyptian children with chronic liver diseases. World Journal of Gastroenterology 13: 785–790. 17278204 10.3748/wjg.v13.i5.785 PMC4066014

[27] Wu HC, Lin CC, Chen WC, Chen HY, Tsai FJ (2003) Osteocalcin gene HindIII C/T polymorphism is a biomarker for prostate cancer and responsiveness to hormone therapy. Eur Urol 43: 197–200. 10.1016/S0302-2838(02)00541-9 12565780

[28] Lu Y, Wu Z, Peng Q, Ma L, Zhang X, et al (2014) Role of IL-4 Gene Polymorphisms in HBV-Related Hepatocellular Carcinoma in a Chinese Population. PLoS One 9: e110061 10.1371/journal.pone.0110061 25295591 PMC4190355

[29] Liu Y, Sui J, Zhai L, Yang S, Huang L, et al (2014) Genetic polymorphisms in hypoxia-inducible factor-1a gene and its association with HBV-related hepatocellular carcinoma in a Chinese population. Med Oncol 31: 200 10.1007/s12032-014-0200-8 25195037

[30] Xi XE, Liu Y, Lu Y, Huang L, Qin X, et al (2015) Interleukin-17A and interleukin-17F gene polymorphisms and hepatitis B virus-related hepatocellular carcinoma risk in a Chinese population. Med Oncol 32: 355 10.1007/s12032-014-0355-3 25429834

[31] Qin X, Deng Y, Liao XC, Mo CJ, Li X, et al (2012) The IL-8 gene polymorphisms and the risk of the hepatitis B virus/infected patients. DNA Cell Biol 31: 1125–1130. 10.1089/dna.2011.1438 22335768

[32] Shi YY, He L (2005) SHEsis, a powerful software platform for analyses of linkage disequilibrium, haplotype construction, and genetic association at polymorphism loci. Cell Res 15: 97–98. 10.1038/sj.cr.7290272 15740637

[33] Qin X, Wu HL, Mo ZN, Chen ZP, Luo SX, et al (2014) Reference Interval for Osteocalcin in Chinese Han Ethnic Males from the Fangchenggang Area Male Health and Examination Survey. Clinical Laboratory 60: 1177–1185. 25134387 10.7754/clin.lab.2013.130331

[34] Price PA, Parthemore JG, Deftos LJ (1980) New biochemical marker for bone metabolism. Measurement by radioimmunoassay of bone GLA protein in the plasma of normal subjects and patients with bone disease. J Clin Invest 66: 878–883. 10.1172/JCI109954 6968755 PMC371521

[35] Ivaska KK, Hentunen TA, Vaaraniemi J, Ylipahkala H, Pettersson K, et al (2004) Release of intact and fragmented osteocalcin molecules from bone matrix during bone resorption in vitro. J Biol Chem 279: 18361–18369. 10.1074/jbc.M314324200 14970229

[36] Strohmaier WL, Schlee-Giehl K, Bichler KH (1996) Osteocalcin response to calcium-restricted diet: a helpful tool for the workup of hypercalciuria. Eur Urol 30: 103–107. 8854076 10.1159/000474153

[37] Marcellini M, De Carli P, Abbolito MR, Mainiero G, Cantiani R (1992) Serum osteocalcin in monitoring bone metastases in advanced prostatic cancer. Eur Urol 21 Suppl 1: 102–104. 1385126 10.1159/000474903

[38] Yeung F, Law WK, Yeh CH, Westendorf JJ, Zhang Y, et al (2002) Regulation of human osteocalcin promoter in hormone-independent human prostate cancer cells. J Biol Chem 277: 2468–2476. 10.1074/jbc.M105947200 11684680

