# Supplementary figures and images for: Relationships between the Osteocalcin Gene Polymorphisms, Serum Osteocalcin Levels, and Hepatitis B Virus-Related Hepatocellular Carcinoma in a Chinese Population
Source: PLoS One. 2015 Jan 14;10(1):e0116479. doi: 10.1371/journal.pone.0116479 (PMC4294662; doi:10.1371/journal.pone.0116479)

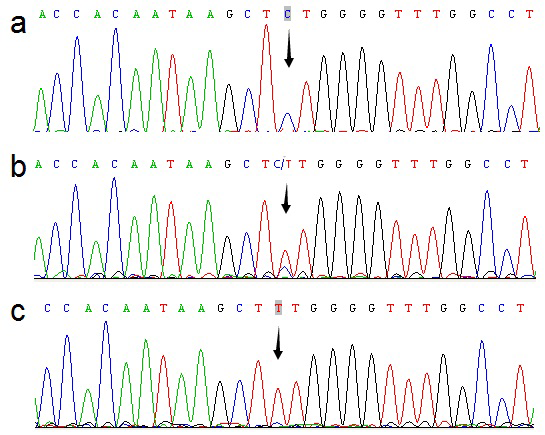

Supplement: S1 Fig — Arrow in parts a–c indicates (hh) CC, (Hh) CT and (HH) TT genotypes, respectively. (TIF) [file pone.0116479.s001.tif]

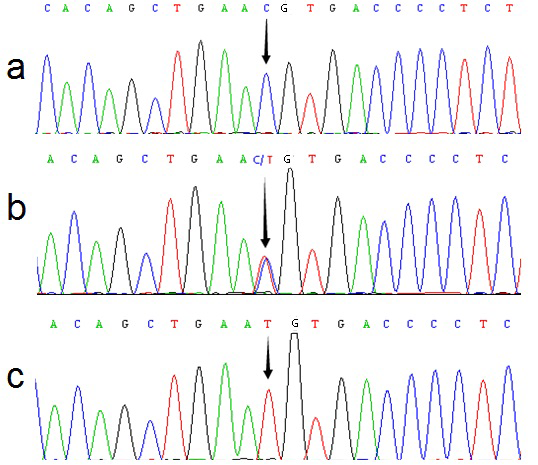

Supplement: S2 Fig — Arrow in parts a–c indicates CC, CT and TT genotypes, respectively. (TIF) [file pone.0116479.s002.tif]
