# Supplementary material for: Relationships between the Osteocalcin Gene Polymorphisms, Serum Osteocalcin Levels, and Hepatitis B Virus-Related Hepatocellular Carcinoma in a Chinese Population
Source: PLoS One. 2015 Jan 14;10(1):e0116479. doi: 10.1371/journal.pone.0116479 (PMC4294662; doi:10.1371/journal.pone.0116479)
Supplement: S1 Table — (DOCX) [file pone.0116479.s003.docx]

| **Table S1.** Comparison of genotype and allele frequencies in the healthy control subjects of our study and that from the HapMap project. | | | | | | | | | | |
| --- | --- | --- | --- | --- | --- | --- | --- | --- | --- | --- |
| Polymorphisms | Individual Group | Samples, N | Genotype frequency, n(%) | | |  |  | Alleles frequency, n(%) | |  |
|  |  |  | TT | CT | CC | P values |  | T | C | P values |
| rs1543297 |  |  |  |  |  |  |  |  |  |  |
| Present study | Asian | 170 | 65 (38.3) | 83 (48.8) | 22 (12.9) | - |  | 213 (62.7) | 127 (37.3) | - |
| [HCB](http://www.ncbi.nlm.nih.gov/pmc/articles/PMC4190355/table/pone-0110061-t004/#nt108) | Asian | 86 | 32 (37.2) | 42 (48.8) | 12 (14.0) | 0.970 |  | 106 (61.6) | 66 (38.4) | 0.822 |
| JPT | Asian | 172 | 76 (44.2) | 84 (48.8) | 12 (7.0) | 0.150 |  | 236 (68.6) | 108 (0.314) | 0.101 |
| [CEU](http://www.ncbi.nlm.nih.gov/pmc/articles/PMC4190355/table/pone-0110061-t004/#nt108) | European | 226 | 6 (2.7) | 76 (33.6) | 144 (63.7) | <0.001 |  | 88 (19.5) | 364 (80.5) | <0.001 |
| [YRI](http://www.ncbi.nlm.nih.gov/pmc/articles/PMC4190355/table/pone-0110061-t004/#nt108) | Sub-Saharan African | 224 | 100 (44.6) | 100 (44.6) | 24 (10.7) | 0.423 |  | 300 (67.0) | 148 (33.0) | 0.208 |

HCB, Han Chinese in Beijing, China; JPT, Japanese in Tokyo, Japan; CEU, Utah residents with northern and western European ancestry; YRI, Yoruba in Ibadan, Nigeria
